# Is Female Wellness Affected When Men Blame Them for Erectile Dysfunction?

**DOI:** 10.1016/j.esxm.2021.100352

**Published:** 2021-05-29

**Authors:** Justin M. Dubin, W. Austin Wyant, Navin C. Balaji, Iakov V. Efimenko, Quinn C. Rainer, Belen Mora, Lisa Paz, Ashley G. Winter, Ranjith Ramasamy

**Affiliations:** 1 Department of Urology, University of Miami Miller School of Medicine, Miami, FL, USA; 2 Department of Urology, Kaiser Permanente, Portland, OR, USA

**Keywords:** Erectile dysfunction, Female sexual dysfunction, Female sexual health, Female wellness, Male blame, Relationships, Sexual health, Wellness

## Abstract

**Introduction:**

Several studies have investigated the association between erectile dysfunction (ED), its treatment, and female sexual dysfunction, but the impact of males blaming their female partners for their ED remains unknown.

**Aims:**

To investigate whether women who are blamed by their male partners for their ED experience worse overall sexual function and satisfaction.

**Methods:**

We performed a global, cross-sectional web-based survey to investigate female perceptions of ED. We distributed the 30-item survey via email, Reddit, Amazon Mechanical Turk, and Facebook. Women 18 years of age or older were eligible to participate and answered questions based on a 5-point Likert scale. Women were grouped by ages 18-29, 30-39, and 40 and older.

**Main Outcome Measures:**

The survey collected data that included general demographics and questions regarding experiencing male blame for ED and its relationship with each subject's sexual health and wellness.

**Results:**

A total of 13,617 females participated in the survey. Of the women surveyed, 79% have experienced their partner losing their erection during sexual activity and approximately 1 out of 7 women (14.7%) had experienced being blamed by their partner for loss of their erection. Women who were blamed for their partner's ED were more likely to end the sexual encounter, were less sexually satisfied, and were more likely to end relationships due to their partner's ED.

**Conclusion:**

Approximately 1 out of 7 women have experienced male blame for their partner's ED which is associated with negative impacts on female mental health, sexual satisfaction and the success of the overall partnership. Because of its widespread impact on female wellness, male blame should be considered during evaluation of female sexual history and men must be educated on the significant impact their reactions during intimacy have on their female partners and their relationships as a whole. **Dubin JM, Wyant WA, Balaji NC, et al. Is Female Wellness Affected When Men Blame Them for Erectile Dysfunction?. Sex Med 2021;9:100352.**

## INTRODUCTION

Male erectile dysfunction (ED), defined as the consistent or recurrent inability to attain or maintain an erection sufficient for sexual activity, is the most common form of sexual dysfunction in men.^1^^,^^2^ A diagnosis of ED is said to affect as high as 52% of the male population with 5-20% of men suffering from moderate to severe ED.^3^ ED's prevalence is strongly correlated with increasing age: ED is seen in 15% of men 40–50 years old, 45% of men in their 60s, and 70% of men over 70 years old.^2^^,^ ^4^ Other variables directly correlated with ED, when adjusting for age, include smoking behavior, heart disease, hypertension, diabetes, indexes of depression and anger, and emotional stress. ^2^^,^^5^ The literature has traditionally focused on how ED affects men, with limited research exploring how ED affects female sexual function.^6^

Female sexual dysfunction (FSD), is often a heterogenous disorder that can take many forms, including diminished arousal, orgasm difficulties, low interest or desire, and dyspareunia. Because of its multitude of incorporated signs and symptoms and varied instruments for diagnosis, there have many inconsistencies in how it has been measured over the years, with many questionnaires like the Female Sexual Function Index (FSFI) being used to indicate the risk of sexual dysfunction in women.^7^^,^ ^8^ For this reason, the prevalence of FSD has been difficult to accurately capture, with some data suggesting individual female sexual disorders ranging from 20.6% to 43.1% with the overall prevalence risk of FSD quoted to be as high as 41%.^9^^,^ ^10^ Although there is inconsistency in the diagnosis of FSD, its causes are known to be both physiological and psychological. Psychological causes include emotional – pertaining to poor body image/self-esteem/stress and relational – marital or relationship problems.^7^^,^ ^11^ These psychological effects on sexual function have been noted in multiple studies: in cases where females are partners of men with ED, studies have found significantly lower sexual activity, diminished sexual relationship satisfaction, and significantly more relationship problems ^12^^,^^13^ The attitudes of females towards their male partner's ED, including its severity and treatment, have also been strongly associated with the likelihood of the male partner mentioning ED to a doctor and trying phosphodiesterase type 5 (PDE5) inhibitor therapy.^6^ Compared to couples that do not use PDE5 inhibitors, women with partners that initiate PDE5 inhibitor therapy experienced significantly more sexual desire, arousal, and orgasm.^12^

Given the emotional and relational causes of FSD, we were interested in whether more specific interactions between couples during intercourse that could affect the psychology of females and thus influence their female sexual function. In couples experiencing ED, the impact of males blaming their female partners for their ED on female sexual functioning has yet to be investigated. We hypothesized that women who are blamed by their male partners for their ED experience worse overall sexual function and satisfaction. Our objective was to explore female perceptions of ED, its role in their relationship with their partner, and the influence of male blame on female sexual health. We planned on accomplishing this by distributing anonymous surveys regarding their sexual health and perceptions of ED to women on a global scale.

## MATERIAL AND METHODS

We performed a global, cross-sectional web-based survey to investigate female perceptions of ED. From April 22 to June 16, 2020, we distributed the 30-item survey via email, Reddit, Amazon Mechanical Turk, and Facebook. The survey was conceptualized, developed, constructed and reviewed by the authors. Due to its novel concept, was not based off of any previously validated surveys. Surveys were distributed with the same IRB approved introduction on all media platforms for consistency purposes. The name published for the survey study during distribution was “Female Perceptions of Erectile Dysfunction.” Using Reddit as a platform, we distributed an online survey to subscribers of various subreddits via a forum post and provided an anonymous link for completion of the survey. Each subreddit was selected based on its description and relevance to the topic. Moderators of each subreddit were contacted prior to conducting the survey and approval was acquired. For Amazon Mechanical Turk, the survey was available for women above the age of 18 located in the United States to complete. For Facebook, distribution of the survey was performed by posted anonymous links to private groups known to have all female members. The project was approved by the associated university's Institutional Review Board.

Women 18 years of age or older were eligible to participate. Women who never engaged in sexual activity with a male partner were excluded from the study. Women were grouped by ages 18–29, 30-39, and 40 and older. The survey collected demographic information including age, sexual orientation, race, and current relationship status. It also collected data on sexual activity, including satisfaction, desire, and impact on quality of life. Questions regarding ED included whether they have experienced ED with a partner, have been blamed for ED, and how ED affected their sexual desire, confidence and sexual activity. Questions like effect of partners loss of erection on confidence and sense of responsibility for partner losing their erection were answered based on 5-point Likert scales.

Statistical analysis was done using MATLAB (2020) (version R2020a, Natick, Massachusetts: The Mathworks Inc). Responses to survey questions were stratified by several factors including relationship status, age, education level, and male partner blame for ED. It must be noted that because we were asking women, not their male partners about ED that we were unable to use the validated International Index of Erectile Function (IIEF-5) to assess ED. In this study, ED is subjective and can be interpreted as a male partner's inability to obtain or maintain an erectile for sexual activity. Relationship status was analyzed by comparing those in marriages, civil unions, and partnerships to all other relationship statuses. We assessed whether answer choices varied with the different factors using chi squared tests of independence. Direct comparisons between groups of interest were made using two proportion Z tests. An alpha of 0.05 was used for all significance testing.

## RESULTS

A total of 13,617 females from 130 different countries participated in the survey. Of the respondents, 9825 (73.7%) were 18 – 29 years old, 2612 (19.6%) were 30 – 39 years old, and 897 (6.7%) were 40 or older. Further demographics can be found on Table 1.

**Table 1:** Demographic data

Variables	Counts	Percentage (%)
**Age**		
18 – 29	9825	73.7
30 – 39	2612	19.6
40 +	897	6.7
**Country (Top 3)**		
USA	8512	62.5
Canada	1156	8.5
United Kingdom	1077	7.9
**Race (Top 3)**		
White / Caucasian	10615	74.5
Asian	1221	8.6
Spanish, Hispanic, Latino	1156	8.2
**Relationship Status**		
Single	2926	22.8
Married	2348	18.3
Monogamous	6582	51.3
Open	360	2.8
Civil Union / Domestic Partnership	293	2.3
Widowed	21	0.2
Divorced	160	1.2
Other	128	1.0

**Figure 1:**
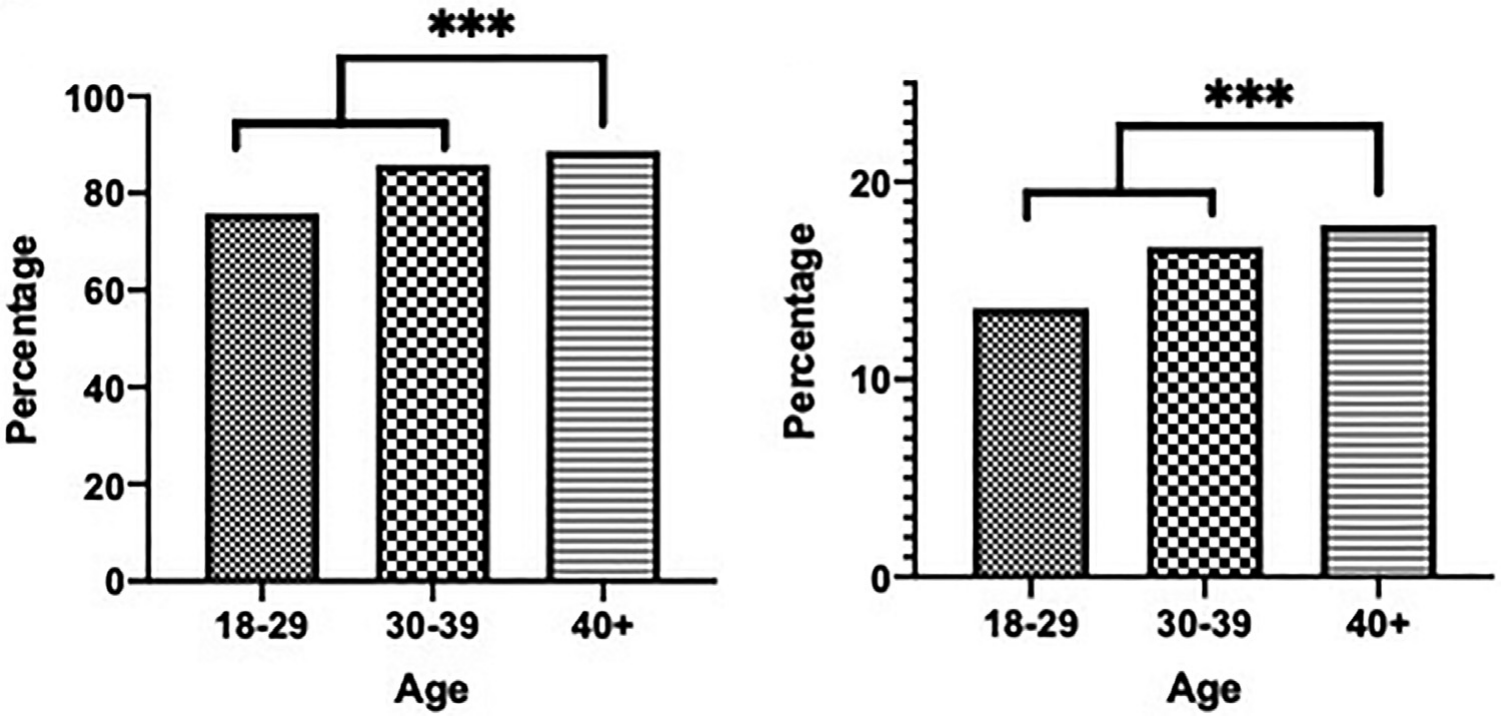
A. Age vs. percentage of women who experienced their partner losing an erection. B. Age vs. percentage of women who were blamed by their partner for their ED.

The majority (87.8%) of the women surveyed were sexually active, with no significant difference in sexual activity rates amongst ages groups (ages 18-29, 30–39, 40 and older -87.11%. 90.7%, and 86.7%, respectively, *P* = .211). The majority of women who were not sexually active were single women (74.7%). Most of the women (91.1%) who desired sexual activity were sexually active, while most women (60.9%) who did not desire sexual activity were not sexually active. Of the women surveyed, 79% have experienced their partner losing their erection during sexual activity and approximately 1 out of 7 women (14.7%) had experienced being blamed by their partner for loss of their erection. When stratified by age - women ages 18–29, ages 30–39, and 40 and above - the majority of women have experienced a partner losing an erection during sexual activity (75.8%, 85.9%, and 88.7%, respectively) and a small but significant amount have been blamed by their partner for their ED (13.6%, 16.7%, and 17.8%, respectively). In both cases, the incidence rate was significantly higher for women above 40 (*P* < .001, *P* < .001). (Figure 1)

**Figure 2:**
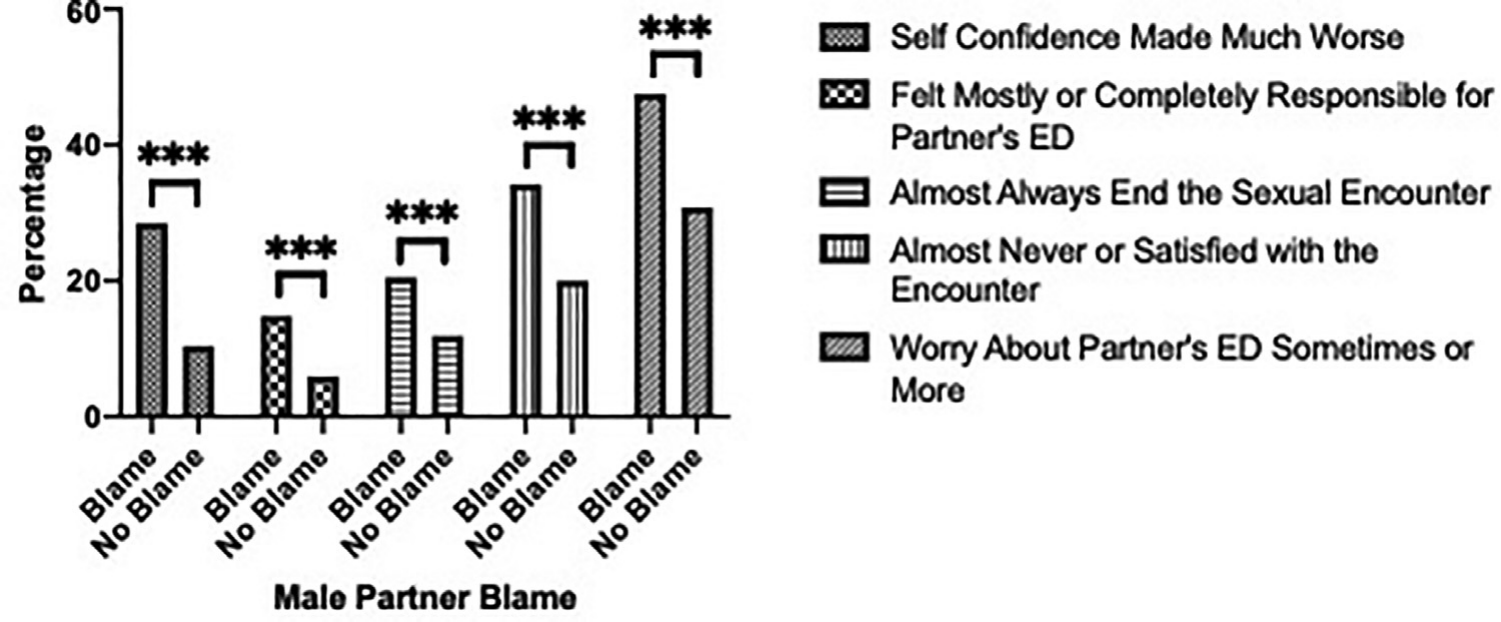
Effect of male blame on female sentiments.

Women who were blamed for their partner's ED experienced significantly worse self-confidence compared to those who were not blamed (*P* < .001). They also felt an increased sense of strong responsibility for their partner's ED and worried more about their partner's ED compared to those who were not blamed (*P* < .001 and *P* < .001, respectively). In addition to their worsened sense of self-confidence, the quality of their sexual encounters was significantly worse. Women blamed for their partner's ED were more likely to almost always end the sexual encounter after ED occurred and were more likely to almost never be satisfied during the sexual encounter (*P* < .001 and *P* < .001, respectively). (Figure 2) In assessing blame's effect on relationships, women blamed for ED were more likely to have ended and were more willing to end future relationships due to their partner's ED (*P* < .001 and *P* < .001, respectively).

**Figure 3:**
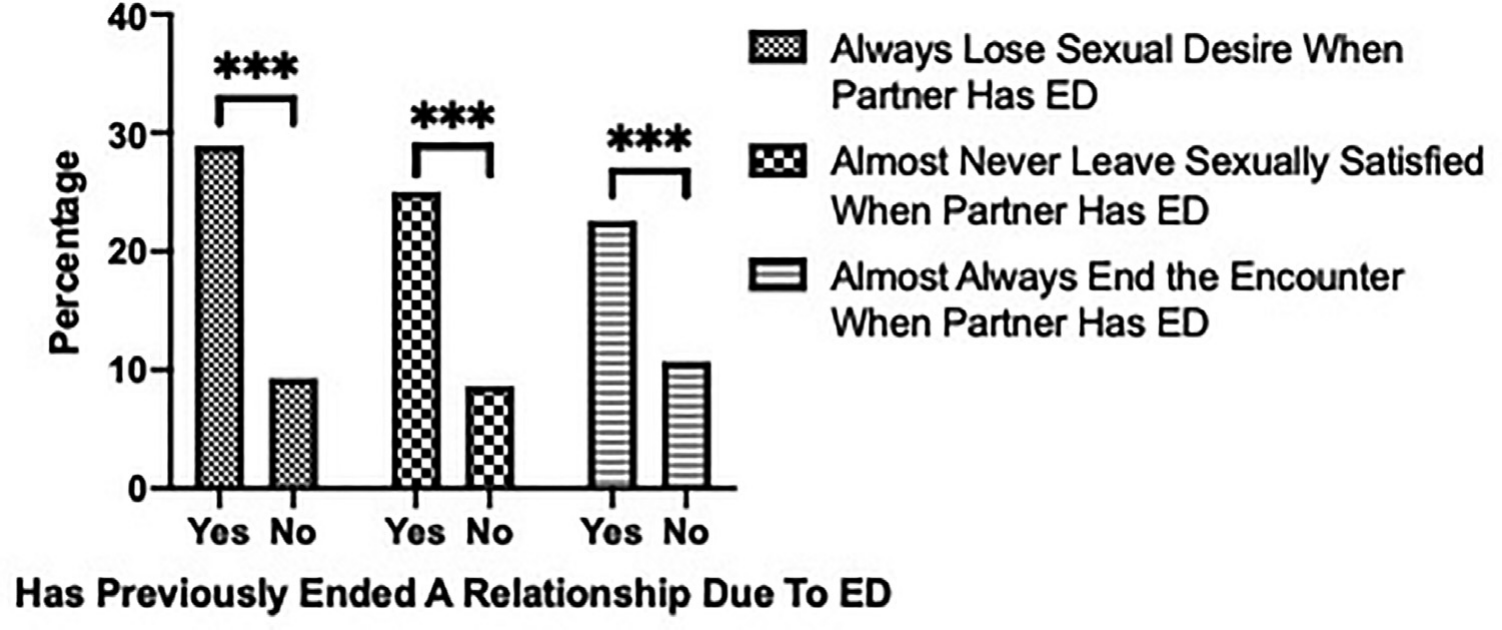
Female desire and satisfaction during sexual encounters involving ED and their effects on relationships.

We also assessed the role of female sexual desire and satisfaction in the face of ED and the effects it has on the overall relationship. Women who always lose their sexual desire when their partner experiences ED are significantly more likely to end their relationship due to ED. In addition, when their male partner has ED, women who almost never leave a sexual encounter satisfied are significantly more likely to end their relationship than women who leave an encounter at least somewhat satisfied (*P* < .001). Likewise, women who almost always end their sexual encounter when their partner experiences ED are significantly more likely to end their relationship due to their partner's ED (*P* < .001) (Figure 3).

## DISCUSSION

Our study explores the role of male blame for ED on their female partner and its potential implications. This is the first study to demonstrate that approximately 1 out of every 7 women have been blamed by their male partner for ED, with increasing prevalence of experiencing blame in women above the age of 40. Even more concerning are the implications of male blame on female wellness. Women who were blamed by their partner for their ED were more likely to experience worse self-confidence, an increased sense of responsibility for their partner's ED, increased worry about their partner's ED and the quality of their sexual encounters were worse. Male blame also impacted the relationship – women who were blamed for ED were more likely to have ended and were more willing to end relationships due to their partner's ED.

Sexual health and satisfaction are important components of patient health and happiness.^14^^,^ ^15^ Previous studies have shown that the majority of men and women are sexually active across all age groups, with some showing a decrease in activity with an increase in subject age.^16^, ^17^, ^18^ However, most of these studies exploring the sexual activity of middle and older aged women were performed over 10 years ago. There has been a significant shift in the focus on both male and female sexual health since then and the high sexual activity rates reported in women above 40 years old in our study reflect that.^19^^,^ ^20^ Based on our data, it is important for physicians to inquire about the sexual activity of all their female patients, not just younger women under the age of 40.

Because FSD can have both emotional and relational etiologies, the sexual relationship between the female and their male partner may be a nidus for the woman's sexual dysfunction, especially when the male suffers from sexual dysfunction themselves.^7^^,^ ^11^ When evaluating sexual health and wellness in women, several studies have focused on the role of their partner's ED.^6^^,^ ^12^ One component that has not previously been explored is the ramification of males blaming their ED on their partner. Data already suggests that body image concerns negatively affect sexual pleasure and promote sexual problems in both men and women.^21^ In moments of sexual intimacy, we wanted to investigate whether the specific event of blame by a male partner could affect women's sexual health and potentially put them at risk for sexual dysfunction. Our study shows that male blame has a significant effect on the psyche and sexual experience of female partners as well as the success of the overall partnership. A recent systematic review demonstrated that significant risk factors for FSD includes poor mental health, stress, and relationship satisfaction.^22^ This study shows that male blame for ED may directly contribute to FSD risk factors. More studies need to confirm this finding. We were not able to report the prevalence of FSD in our participants, as we did not include the FSFI in our survey. Future studies should explicitly explore FSD prevalence in those women experiencing male blame.

More concerning, male blame is quite prevalent. With approximately 1 out of every 7 women experiencing it and no previous data ever exploring the topic, physicians must make sure to educate female patients on the causes of ED and provide an open ear and more importantly, reassurance to those women experiencing it. Therefore, a thorough female sexual history for patients should always include their male partner's sexual function (if applicable) and whether they have been blamed for any sexual issues within the relationship.

An important consideration in the role of male blame in female health and wellness is that although blame affects the female, it is actually the male partner and their reaction to their own sexual dysfunction that potentiates issues for their female partner. The majority of the participants in our study were aged 18 to 29, and although we lack any information on the ages of partners, we can assume most are under the age of 40. In this younger male age group, a prevalence of ED of 79% is high. We are unable to conclude why the prevalence of ED is so high in our study, but based on this male age population, we can only speculate that most of the ED is due to psychogenic causes like anxiety, depression, anger, and partner-related difficulties.^2^^,^ ^23^ These additional stressors that may be causing the male partner's ED may be also influencing the overall relationship as well. Our study stresses the significance of assessing the male partner's overall health and mental wellbeing in any female who is complaining of sexual or psychological issues. Furthermore, educating men on how their reactions to their ED during intimacy strongly influences their female partner's mental health, sexual satisfaction, and their overall relationship may prevent further issues with male blame and strengthen relationships. Only by making men aware of the impact that their actions have on their partner can we potentially correct this preventable factor for FSD.

Limitations of our study potentially includes the method of distribution of our survey which included Amazon Mechanical Turk, Facebook, and Reddit. It is possible that participants were not female users and provided inaccurate information. This is not the first study to utilize Reddit as a source for survey data acquisition and one previous study assessing the validity of Reddit survey responses suggests that the larger pool of participants in such an open forum such as Reddit may potentially compromise the quality of the data.^24^^,^ ^25^ In an attempt to reduce falsification of data by survey participants, our survey was designed with a logic flow algorithm which would immediately end the survey if a particular response did not result in a participant who met our study's criteria. In addition, the Qualtrics survey platform prevents participants from completing the survey more than once on the same IP address. Regarding Amazon Mechanical Turk, there have been previous studies published that have utilized the platform, including at least two in urological journals.^26^^,^ ^27^ Other limitations include the use of less scientific wording to define ED. We used “losing an erection” as a descriptive term in the survey which is not inherently equal to ED, however, we felt that putting in more layman's terms would be easier for subjects to understand and therefore best answer the questions. In addition, factors such as ED severity and female personal information including social status, mental health status, and history of chronic diseases which can affect sexual health were not obtained. Despite these potential limitations, the distribution method for our study provided us with a very large sample size, which was one of the major strengths of our study.

## CONCLUSIONS

Approximately 1 out of 7 women experience male blame for their partner's ED. Male blame is associated with negative impacts on female mental health, sexual satisfaction and the success of the overall partnership. Because of its large impact on multiple aspects of female wellness, male blame may be considered a new novel factor in assessing FSD and should be included in the sexual history taking of all women. Finally, it is important men are educated on the significant impact their reactions during intimacy have on their female partners and their relationships as a whole.

## STATEMENT OF AUTHORSHIP

All authors of this manuscript played an important role in the study from its conceptualization, execution, data analysis, to the writing and revisions of this manuscript according to the CRediT system.

## Appendix Supplementary materials

Image, application 1
