# Supplementary material for: Is Female Wellness Affected When Men Blame Them for Erectile Dysfunction?
Source: Sex Med. 2021 May 29;9(3):100352. doi: 10.1016/j.esxm.2021.100352 (PMC8240332; doi:10.1016/j.esxm.2021.100352)
Supplement: Supplementary file 1 [file mmc1.docx]

Female Perceptions of Erectile Dysfunction

Start of Block: Main Question Block

Q1 (Intro to survey – removed)

| Page Break |  |
| --- | --- |

Q2 What was your assigned sex at birth?

- Male (1)
- Female (2)
- Other (3) ________________________________________________

Skip To: End of Block If What was your assigned sex at birth? != Female

Q3
Age (Years)

▼ 18-29 (1) ... >80 (6)

Q4 What gender do you identify as?

- Male (1)
- Female (2)
- Other (3) ________________________________________________
- Prefer not to answer (4)

| 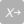 |
| --- |

Q5 In which country do you currently reside?

▼ Afghanistan (1) ... Zimbabwe (1357)

Display This Question:

If List of Countries = United States of America

Q6 In which state do you currently reside?

▼ Alabama (1) ... I do not reside in the United States (53)

Q7 What is the highest level of school you have completed or the highest degree you have received?

- Less than high school degree (1)
- High school graduate (high school diploma or equivalent including GED) (2)
- Some college but no degree (3)
- Associate degree in college (2-year) (4)
- Bachelor's degree in college (4-year) (5)
- Master's degree (6)
- Doctoral degree (e.g. PhD) (7)
- Law degree (JD) (8)
- Medical degree (MD or DO) (9)

Q8 At what level of school did you have a sexual education class (check all that apply)?

- Elementary school (1)
- Middle school (2)
- High school (3)
- College (4)
- Post graduate (5)
- ⊗Never (6)

Q9 What other sources have you used for sexual education (check all that apply)?

- Family member(s) (1)
- Magazine(s) (2)
- Internet search(es) (3)
- Pornography (4)
- TV/Movie(s) (5)
- Podcast(s) (6)
- Medical doctor(s) (7)
- Friend(s) (8)
- Other (please describe) (9) ________________________________________________
- ⊗None (10)

| 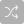 |
| --- |

Q10 Which of the following best describes your sexual orientation?

- Heterosexual (straight) (1)
- Homosexual (lesbian/gay) (2)
- Bisexual (3)
- Other (4) ________________________________________________
- Prefer not to answer (5)

Q11 Choose one or more races that you consider yourself to be:

- White or Caucasian (1)
- Black or African American (2)
- American Indian or Alaska Native (3)
- Asian (4)
- Native Hawaiian or Pacific Islander (5)
- Spanish, Hispanic, or Latino (6)
- Other (7) ________________________________________________
- ⊗Prefer not to answer (8)

| 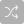 |
| --- |

Q12 What is your current relationship status?

- Single (1)
- Married (2)
- In a monogamous relationship (3)
- In an open relationship (4)
- In a civil union/domestic partnership (5)
- Widowed (6)
- Divorced (7)
- Other (8) ________________________________________________

Q13 Are you currently sexually active (have engaged in sexual activity in the last 6 months)?

- Yes (1)
- No (2)

Q14 Are you currently interested in sexual activity?

- Yes (1)
- No (2)
- Unsure (3)

Q15 Does sexual activity (or lack thereof) currently play an important role in your overall quality of life?

- Not important at all (1)
- Slightly important (2)
- Neutral (3)
- Moderately important (4)
- Very important (5)

Q16 Have you ever engaged in sexual activity with a male partner?

- Yes (1)
- No (2)

Display This Question:

If Are you currently sexually active (have engaged in sexual activity in the last 6 months)? = Yes

And Have you ever engaged in sexual activity with a male partner? = Yes

Q17 How satisfied are you with your male partner’s current sexual function?

- Very satisfied (1)
- Moderately Satisfied (2)
- Neutral (3)
- Slightly satisfied (4)
- Not satisfied at all (5)
- Not applicable (6)

Q18 In general, how much does your male partner's sexual function impact your quality of life?

- Insignificant impact (1)
- Minor impact (2)
- Neutral (3)
- Moderate impact (4)
- Very significant impact (5)
- Not applicable (6)

Q19 Have you ever ended a relationship at least partly due to your male partner having erectile dysfunction (ED)?

- Yes (1)
- No (2)
- Not applicable (3)

Q20 Would you ever end a relationship, at least partly, due to your male partner having erectile dysfunction (ED)?

- Yes (1)
- No (2)
- Not applicable (3)

| 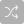 |
| --- |

Q21 Which of the following things, if any, do you think can cause a male to lose their erection (check all that apply)?

- Alcohol (1)
- Prescribed medications (2)
- Recreational drugs (3)
- Male is uncomfortable and/or stressed (4)
- Using a condom or other barrier contraceptives (5)
- Cardiovascular disease (6)
- Older age (7)
- Diabetes (8)
- Surgery (9)
- Male is not attracted to their partner and/or is attracted to someone else (10)
- Pornography use (11)
- Other (12) ________________________________________________

Q22 Do you think that the female partner can be a cause for male erectile dysfunction?

- Yes (1)
- No (2)
- Unsure (3)

Display This Question:

If Do you think that the female partner can be a cause for male erectile dysfunction? != No

Q23 How do you think a female can cause male erectile dysfunction (check all that apply)?

- The woman is not physically attractive to the man (1)
- The woman said something that the man did not like (2)
- The woman did not want to engage in a specific sexual act (3)
- Unsure (4)
- Other (5) ________________________________________________

Display This Question:

If Have you ever engaged in sexual activity with a male partner? = Yes

Q24 Has a male partner ever lost their erection while engaging in sexual activity with you?

- Yes (1)
- No (2)
- Unsure (3)

Display This Question:

If Has a male partner ever lost their erection while engaging in sexual activity with you? = Yes

Q25 Overall, to what degree do you think that you were responsible for your male partner(s) losing their erection(s)?

- Completely responsible (1)
- Mostly responsible (2)
- Unsure (3)
- Somewhat responsible (4)
- Not at all responsible (5)

Display This Question:

If Has a male partner ever lost their erection while engaging in sexual activity with you? = Yes

Q26 Has a male partner who lost their erection ever blamed it on you?

- Yes (1)
- No (2)

Display This Question:

If Has a male partner ever lost their erection while engaging in sexual activity with you? = Yes

Q27 Overall, how has your partner losing their erection affected your confidence?

- Made it much worse (1)
- Made it somewhat worse (2)
- Not affected (3)
- Made it somewhat better (4)
- Made it much better (5)

Display This Question:

If Have you ever engaged in sexual activity with a male partner? = Yes

Q28 How often do you worry about male partners not being able to maintain an erection when engaging in sexual activity with you?

- Never (1)
- Rarely (2)
- Sometimes (3)
- Often (4)
- Always (5)

Display This Question:

If Has a male partner ever lost their erection while engaging in sexual activity with you? = Yes

Q29 Does your male partner’s erectile dysfunction (ED) affect your sexual desire?

- I almost always lose my sexual desire when my partner has ED (1)
- I sometimes lose my sexual desire when my partner has ED (2)
- There is no change in my sexual desire when my partner has ED (3)
- I sometimes have increased sexual desire when my partner has ED (4)
- I almost always have increased sexual desire when my partner has ED (5)

Display This Question:

If Has a male partner ever lost their erection while engaging in sexual activity with you? = Yes

Q30 When your partner has experienced erectile dysfunction during a sexual encounter, how often do you and your partner continue engaging in sexual activity?

- We almost always continue all sexual activity during the encounter (1)
- We usually continue all sexual activity during the encounter (2)
- We occasionally continue all sexual activity during the encounter (3)
- We usually stop all sexual activity during the encounter (4)
- We almost always stop all sexual activity during the encounter (5)

Display This Question:

If Has a male partner ever lost their erection while engaging in sexual activity with you? = Yes

Q31 When your male partner has erectile dysfunction, how often do you end the sexual encounter sexually satisfied?

- Almost always sexually satisfied (1)
- Usually sexually satisfied (2)
- Occasionally sexually satisfied (3)
- Usually not sexually satisfied (4)
- Almost never sexually satisfied (5)

End of Block: Main Question Block

Start of Block: MTurk Number

Q32
If you came to this survey from MTurk, please follow the instructions below. Otherwise, please click the next button to submit your survey.


Here is your ID: ${e://Field/Random%20ID}


Copy this value to paste into MTurk.


When you have copied this ID, please click the next button to submit your survey.

End of Block: MTurk Number
